# Refined quadratic estimations of Shafer’s inequality

**DOI:** 10.1186/s13660-017-1312-4

**Published:** 2017-02-08

**Authors:** Yusuke Nishizawa

**Affiliations:** General Education, Ube National College of Technology, Tokiwadai 2-14-1, Ube, Yamaguchi 755-8555 Japan

**Keywords:** 26D15, 42A10, Shafer’s inequality, an upper bound for arctangent, a lower bound for arctangent

## Abstract

We establish an inequality by quadratic estimations; the double inequality

$$ \frac{\pi^{2} x}{4 +\sqrt{(\pi^{2} -4)^{2} + (2\pi x)^{2}}} < \arctan{x} < \frac{\pi^{2} x}{4 +\sqrt{32+ (2\pi x)^{2}}} $$

holds for $x>0$, where the constants $(\pi^{2} -4)^{2}$ and 32 are the best possible.

## Introduction

Shafer [1–3] showed that the inequality

1.1 $$ \arctan{x} > \frac{8 x}{3 +\sqrt{25+ \frac{80}{3} x^{2}}} $$

holds for $x>0$. Various Shafer-type inequalities are known, and they have been applied, extended and refined, see [4–8] and [9–12]. Especially, Zhu [12] showed an upper bound for inequality (1.1) and proved that the following double inequality

1.2 $$ \frac{8 x}{3 +\sqrt{25 + \frac{80}{3}x^{2}}} < \arctan{x} < \frac{8 x}{3 +\sqrt{25+ \frac{256}{\pi^{2}} x^{2}}} $$

holds for $x>0$, where the constants $80/3$ and $256/\pi^{2}$ are the best possible. Recently, in [8], Sun and Chen proved that the following inequality

1.3 $$ \arctan{x}< \frac{8 x+\frac{32}{4725}x^{7}}{3 +\sqrt{25+ \frac{80}{3} x^{2}}} $$

holds for $x>0$; moreover, they showed that the inequality

1.4 $$ \frac{8 x+\frac{32}{4725}x^{7}}{3 +\sqrt{25+ \frac{80}{3} x^{2}}} < \frac{8 x}{3 +\sqrt{25+ \frac{256}{\pi^{2}} x^{2}}} $$

holds for $0< x< x_{0} \cong 1.4243$. In this paper, we shall establish the refinements of inequalities (1.2) and (1.3).

## Results and discussion

Motivated by (1.2), (1.3) and (1.4), in this paper, we give inequalities involving arctangent. The following are our main results.

### Theorem 2.1

*For* $x>0$, *we have*

2.1 $$ \frac{\pi^{2} x}{4 +\sqrt{(\pi^{2} -4)^{2} + (2\pi x)^{2}}} < \arctan{x} < \frac{\pi^{2} x}{4 +\sqrt{32+ (2\pi x)^{2}}} , $$

*where the constants* $(\pi^{2} -4)^{2}$ *and* 32 *are the best possible*.

### Theorem 2.2

*For* $x> \alpha $, *we have*

2.2 $$ \frac{\pi^{2} x}{4 +\sqrt{(\pi^{2} -4)^{2} + (2\pi x)^{2}}} >\frac{8 x}{3 +\sqrt{25 + \frac{80}{3} x^{2}}} , $$

*where the constant* $\alpha = \sqrt{\frac{9600 -1860 \pi^{2} +90 \pi^{4}}{2304 -480 \pi^{2} +25 \pi^{4}}} \cong 2.26883$ *is the best possible*.

### Theorem 2.3

*For* $x> \beta$, *we have*

2.3 $$ \frac{8 x}{3 +\sqrt{25 + \frac{256}{\pi^{2}} x^{2}}} >\frac{\pi^{2} x}{4 +\sqrt{32 + (2\pi x)^{2}}} , $$

*where the constant* $\beta =\sqrt{ \frac{4096 +1536 \pi^{2} -528 \pi^{4} +24 \pi^{6} +\pi^{8}}{4096 \pi^{2} -768 \pi^{4} +36 \pi^{6}}} \cong 1.30697$ *is the best possible*.

### Theorem 2.4

*For* $x> \gamma$, *we have*

2.4 $$ \frac{8 x+\frac{32}{4725}x^{7}}{3 +\sqrt{25+ \frac{80}{3} x^{2}}} >\frac{\pi^{2} x}{4 +\sqrt{32 + (2\pi x)^{2}}} , $$

*where the constant* $\gamma \cong 1.38918$ *is the best possible and satisfies the equation*

$$\begin{aligned} &151200 -14175 \pi^{2} + 128 \gamma^{6} - 1575 \sqrt{15} \pi^{2} \sqrt{15 + 16 \gamma^{2}} \\ &\quad {}+ 75600 \sqrt{8 + \pi^{2} \gamma^{2}} + 64 \gamma^{6} \sqrt{8 + \pi^{2} \gamma^{2}}=0. \end{aligned}$$

From Theorems 2.1, 2.2, 2.3 and 2.4, we can get the following proposition, immediately.

### Proposition 2.5

*The double inequality* (2.1) *is sharper than* (1.2) *for* $x > \alpha$. *Moreover*, *the right*-*hand side of* (2.1) *is sharper than* (1.3) *for* $x>\gamma$.

### Proof of Theorem 2.1

Becker-Stark’s inequality is known as the inequality

2.5 $$ \frac{8}{\pi^{2} -4x^{2}} < \frac{\tan{x}}{x} < \frac{\pi^{2}}{\pi^{2} -4x^{2}} $$

which holds for $0< x < \pi/2$. Also, Becker-Stark’s inequality (2.5) has various applications, extensions and refinements, see [13–16] and [17–19]. Especially, Zhu [19] gave the following refinement of (2.5): The inequality

2.6 $$\begin{aligned} \frac{8}{\pi^{2} -4 x^{2}} + \frac{2}{\pi^{2}} -\lambda \bigl( \pi^{2} -4 x^{2}\bigr) < \frac{\tan{x}}{x} < \frac{8}{\pi^{2} -4 x^{2}} +\frac{2}{\pi^{2}} -\mu \bigl(\pi^{2} -4 x^{2}\bigr) \end{aligned}$$

holds for $0< x<\pi/2$, where the constants $\lambda = (\pi^{2} -9)/(6\pi^{4})$ and $\mu = (10 -\pi^{2} )/\pi^{4}$ are the best possible. In this paper, the result of Zhu (2.6) plays an important role in the proof of Theorem 2.1.

#### Proof of Theorem 2.1

The equation

$$\begin{aligned} \arctan{x} = \frac{\pi^{2} x}{4 +\sqrt{c+ (2 \pi x)^{2}}} \end{aligned}$$

is equivalent to

$$\begin{aligned} c= \frac{\pi^{4} x^{2} -8 \pi^{2} x \arctan{x} +16 \arctan^{2} {x} -4 \pi^{2} x^{2} \arctan^{2} {x}}{\arctan^{2} {x}} . \end{aligned}$$

We set $t= \arctan{x}$, then

$$\begin{aligned} c &= \frac{\pi^{4} \tan^{2}{t}}{t^{2}} -\frac{8 \pi^{2} \tan{t}}{t} +16 -4 \pi^{2} \tan^{2}{t} \\ &= 16 +F_{1}(t) . \end{aligned}$$

First, we assume that $0< t\leq 1/2$. Here, the derivative of $F_{1}(t)$ is

$$\begin{aligned} F_{1}'(t) &= -\frac{8 \pi^{2} \sec^{2}{t}}{t} +\frac{8 \pi^{2} \tan{t}}{t^{2}} -8 \pi^{2} \sec^{2}{t} \tan{t} +\frac{2 \pi^{4} \sec^{2}{t} \tan{t}}{t^{2}} - \frac{2 \pi^{4} \tan^{2}{t}}{t^{3}} \\ &= \frac{\sin{t}}{\cos^{2}{t}} \biggl( -\frac{8 \pi^{2} }{t \sin{t}} +\frac{8 \pi^{2} \cos{t}}{t^{2}} - \frac{8 \pi^{2}}{\cos{t}} +\frac{2 \pi^{4}}{t^{2} \cos{t}} -\frac{2 \pi^{4} \sin{t}}{t^{3}} \biggr) \\ &= \frac{\sin{t}}{\cos^{2}{t}} F_{2}(t) . \end{aligned}$$

Since we have

$$\begin{aligned} t-\frac{t^{3}}{6} < \sin{t} < t-\frac{t^{3}}{6}+\frac{t^{5}}{120} \end{aligned}$$

and

$$\begin{aligned} 1 -\frac{t^{2}}{2} +\frac{t^{4}}{24} -\frac{t^{6}}{720} < \cos{t} < 1 - \frac{t^{2}}{2} +\frac{t^{4}}{24} \end{aligned}$$

for $0 < t <\pi/2$, the following inequality holds:

$$\begin{aligned} F_{2}(t) &< -\frac{8 \pi^{2}}{t (t-\frac{t^{3}}{6} +\frac{t^{5}}{120} )} +\frac{8 \pi^{2} ( 1 -\frac{t^{2}}{2} +\frac{t^{4}}{24} )}{t^{2}} \\ & \quad {}-\frac{8 \pi^{2}}{ (1-\frac{t^{2}}{2}+\frac{t^{4}}{24} )} +\frac{2 \pi^{4}}{t^{2} ( 1 -\frac{t^{2}}{2} +\frac{t^{4}}{24} -\frac{t^{6}}{720} )} -\frac{2 \pi^{4} (t-\frac{t^{3}}{6} )}{t^{3}} \\ &= \frac{\pi^{2} F_{3}(t)}{3 (120-20 t^{2}+t^{4} ) (24-12 t^{2}+t^{4} ) (-720+360 t^{2}-30 t^{4}+t^{6} )} , \end{aligned}$$

where $F_{3}(t) = 82944000-8294400 \pi^{2} -72990720 t^{2} +7084800 \pi^{2} t^{2} +24883200 t^{4} -2246400 \pi^{2} t^{4} -4832640 t^{6} +371520 \pi^{2} t^{6} +596736 t^{8} -35904 \pi^{2} t^{8} -48192 t^{10} +2076 \pi^{2} t^{10} +2472 t^{12} -68 \pi^{2} t^{12} -74 t^{14} +\pi^{2} t^{14} +t^{16}$. We set $s=t^{2}$, then

$$\begin{aligned} F_{3}(t) &> 82944000-8294400 \biggl( \frac{315}{100} \biggr)^{2} -72990720 s+7084800 \biggl( \frac{314}{100} \biggr)^{2} s \\ & \quad {}+24883200 s^{2}-2246400 \biggl( \frac{315}{100} \biggr)^{2} s^{2}-4832640 s^{3}+371520 \biggl( \frac{314}{100} \biggr)^{2} s^{3} \\ &\quad {} +596736 s^{4}-35904 \biggl( \frac{315}{100} \biggr)^{2} s^{4}-48192 s^{5}+2076 \biggl( \frac{314}{100} \biggr)^{2} s^{5} \\ &\quad {} +2472 s^{6}-68 \biggl( \frac{315}{100} \biggr)^{2} s^{6}-74 s^{7}+ \biggl( \frac{314}{100} \biggr)^{2} s^{7}+s^{8} \\ & = 642816-\frac{78435648 s}{25}+2593296 s^{2} -\frac{146200176 s^{3}}{125} + \frac{6011964 s^{4}}{25} \\ &\quad {} -\frac{17327169 s^{5}}{625} +\frac{179727 s^{6}}{100}-\frac{160351 s^{7}}{2500}+s^{8} \\ & = \frac{1}{2500} \bigl(1607040000 -7843564800 s +6483240000 s^{2} -2924003520 s^{3} \\ &\quad {} +601196400 s^{4} -69308676 s^{5} +4493175 s^{6} -160351 s^{7} +2500 s^{8}\bigr) \\ & = \frac{1}{2500} \biggl( 1607040000 -7843564800 s + \biggl( \frac{7}{8} \biggr) 6483240000 s^{2} \\ &\quad {} + s^{2} \biggl( \biggl( \frac{1}{8} \biggr) 6483240000 -2924003520 s +601196400 s^{2} -69308676 s^{3} \biggr) \\ &\quad {} +s^{6}\bigl(4493175 -160351 s +2500 s^{2}\bigr) \biggr) \\ & = \frac{1}{2500} \bigl( F_{4}(s) + s^{2} F_{5}(s) +s^{6} F_{6}(s) \bigr) . \end{aligned}$$

We shall show that the functions $F_{4}(s)>0$, $F_{5}(s)>0$ and $F_{6}(s)>0$. Here,

$$\begin{aligned} F_{4}(s) &= 5400 \bigl(297600-1452512 s+1050525 s^{2} \bigr) \\ & =5400 F_{7}(t) . \end{aligned}$$

The derivative of $F_{7}(t)$ is

$$\begin{aligned} F'_{7}(s) &= 2( -726256 +1050525s) \\ &\leq 2 \biggl( -726256 +1050525 \biggl( \frac{1}{4} \biggr) \biggr) \\ &= -\frac{1854499}{2} . \end{aligned}$$

Since $F_{7}(s)$ is strictly decreasing for $0< s< 1/4$ and $F_{7}(1/4) = 2077/16$, we have $F_{4}(s) >0$.

$$\begin{aligned} F_{5}(s) &= 36 \bigl(22511250-81222320 s+16699900 s^{2}-1925241 s^{3}\bigr) \\ &> 36 \bigl(22511250-81222320 s -1925241 s^{3}\bigr) \\ &\geq 36 \biggl( 22511250-81222320 \biggl( \frac{1}{4} \biggr) -1925241 \biggl( \frac{1}{4} \biggr)^{3} \biggr) \\ &= \frac{1854335151}{16} \end{aligned}$$

and

$$\begin{aligned} F_{6}(s) &> 4493175-160351 \biggl( \frac{1}{4} \biggr) \\ &= \frac{17812349}{4} . \end{aligned}$$

Therefore, we can get $F_{3}(t)>0$. By $120-20 t^{2}+t^{4}>0$, $24-12 t^{2}+t^{4}>0$ and $-720+360 t^{2}-30 t^{4}+t^{6}<0$, thus $F_{2}(t) <0$ and $F_{1}(t)$ is strictly decreasing for $0 < t <1/2$. From $F_{1}(0+) = (\pi ^{2} -4 )^{2}-16$, we can get

$$\begin{aligned} F_{1} \biggl( \frac{1}{2} \biggr) \leq F_{1}(t) < \bigl(\pi ^{2} -4 \bigr)^{2} -16 \end{aligned}$$

for $0< t \leq 1/2$. Next, we assume that $1/2 < t < \pi/2$. From inequality (2.6), we have

$$\begin{aligned} &-8 \pi^{2} \biggl\{ \frac{2}{\pi^{2}} +\frac{8}{\pi^{2} -4 t^{2}} - \frac{ (10 -\pi^{2} ) (\pi^{2} -4 t^{2} )}{\pi^{4}} \biggr\} \\ &\qquad {} +\pi^{2} (\pi -2 t) (\pi +2 t) \biggl\{ \frac{2}{\pi^{2}} + \frac{8}{\pi^{2} -4 t^{2}} -\frac{ (-9 +\pi^{2} ) (\pi^{2} -4 t^{2} )}{6 \pi^{4}} \biggr\} ^{2} \\ &\quad < F_{1}(t) \\ &\quad < -8 \pi^{2} \biggl\{ \frac{2}{\pi^{2}} +\frac{8}{\pi^{2} -4 t^{2}} - \frac{ (-9 +\pi^{2} ) (\pi^{2}-4 t^{2} )}{6 \pi^{4}} \biggr\} \\ &\qquad {}+\pi^{2} (\pi -2 t) (\pi +2 t) \biggl\{ \frac{2}{\pi^{2}} + \frac{8}{\pi^{2} -4 t^{2}} -\frac{ (10 -\pi^{2} ) (\pi^{2} -4 t^{2} )}{\pi^{4}} \biggr\} ^{2} \end{aligned}$$

and

$$\begin{aligned} \frac{G_{1}(t)}{36\pi^{6}} < F_{1}(t) < \frac{G_{2}(t)}{3 \pi^{6}} , \end{aligned}$$

where

$$\begin{aligned} G_{1}(t)& = 4761\pi^{6} -426 \pi^{8} + \pi^{10}-18252\pi^{4} t^{2} +1944\pi^{6} t^{2} -12 \pi^{8} t^{2} \\ &\quad {} +7344\pi^{2} t^{4} -1248\pi^{4} t^{4} +48\pi^{6} t^{4} -5184 t^{6} +1152\pi^{2} t^{6} -64\pi^{4} t^{6} \end{aligned}$$

and

$$\begin{aligned} G_{2}(t) &= -276 \pi^{6} +4 \pi^{8} +3 \pi^{10} -624 \pi^{4} t^{2} +416 \pi^{6} t^{2} -36 \pi^{8} t^{2} \\ &\quad {} +12480 \pi^{2} t^{4} -2688 \pi^{4} t^{4} +144 \pi^{6} t^{4}-19200 t^{6}+3840 \pi^{2} t^{6}-192 \pi^{4} t^{6} . \end{aligned}$$

We set $s=t^{2}$, then

$$\begin{aligned} G_{1}(t) &= 4761 \pi^{6} -426 \pi^{8} + \pi^{10} -12 \pi^{4} \bigl(1521 -162 \pi^{2} + \pi^{4} \bigr) s \\ &\quad {} +48 (-3+\pi ) \pi^{2} (3+\pi ) \bigl(-17 +\pi^{2} \bigr) s^{2} -64 (-3+\pi )^{2} (3 +\pi)^{2} s^{3} \\ &= G_{3}(s) \end{aligned}$$

and

$$\begin{aligned} G_{2}(t) &= -276 \pi^{6} +4 \pi^{8} +3 \pi^{10}-4 \pi^{4} \bigl(156 -104 \pi^{2} +9 \pi^{4} \bigr)s \\ &\quad {} +48 \pi^{2} \bigl(\pi^{2} -10 \bigr) \bigl(-26+3 \pi^{2} \bigr) s^{2} -192 \bigl(\pi^{2} -10 \bigr)^{2} s^{3} \\ &= G_{4}(s) . \end{aligned}$$

The derivatives of $G_{3}(s)$ are

$$\begin{aligned} G'_{3}(s) &= 12\bigl(-1521 \pi^{4} +162 \pi^{6} -\pi^{8} +1224 \pi^{2} s -208 \pi^{4} s \\ &\quad {} +8 \pi^{6} s -1296 s^{2} +288 \pi^{2} s^{2} -16 \pi^{4} s^{2}\bigr) \end{aligned}$$

and

$$\begin{aligned} G''_{3}(t) = 96 (-3+\pi ) (3+\pi ) \bigl(-17 \pi^{2} +\pi^{4} +36 s -4 \pi^{2} s \bigr) . \end{aligned}$$

From the inequality

$$\begin{aligned} -17 \pi ^{2}+\pi ^{4} +\bigl(36 -4 \pi ^{2}\bigr) s & < -17 \pi ^{2}+\pi ^{4} +\bigl(36 -4 \pi ^{2} \bigr) \biggl( \frac{1}{4} \biggr) \\ & = 9-18 \pi ^{2}+\pi ^{4} \\ & \cong -71.2438 , \end{aligned}$$

$G''_{3}(s)<0$ and $G'_{3}(s)$ is strictly decreasing for $1/4 < s < \pi^{2}/4$. Since $G'_{3}(1/4) = 12(-81 +324 \pi^{2}-1574 \pi^{4} +164 \pi^{6} -\pi^{8}) \cong -24310.3$, $G'_{3}(s)<0$ and $G_{3}(s)$ is strictly decreasing for $1/4 < s < \pi^{2}/4$. Therefore, we have $G_{1}(t) > G_{3}(\pi^{2}/4) =576 \pi^{6}$ for $1/2< t< \pi/2$. Next, the derivatives of $G_{4}(s)$ are

$$\begin{aligned} G'_{4}(s)& =4\bigl(-156 \pi^{4} +104 \pi^{6}-9 \pi^{8}+6240 \pi^{2} s-1344 \pi^{4} s+72 \pi^{6} s \\ &\quad {} -14400 s^{2} +2880 \pi^{2} s^{2} -144 \pi^{4} s^{2}\bigr) \end{aligned} $$

and

$$\begin{aligned} G''_{4}(s) = 96 \bigl( 10 - \pi^{2} \bigr) \bigl( 26 \pi^{2} -3 \pi^{4} -120 s +12 \pi^{2} s \bigr) . \end{aligned}$$

From the inequality

$$\begin{aligned} 26 \pi^{2} -3 \pi^{4} -120 s +12 \pi^{2} s & < 26 \pi^{2} -3 \pi^{4} +\bigl(-120 +12 \pi^{2}\bigr) \biggl( \frac{1}{4} \biggr) \\ & = -30+29 \pi ^{2}-3 \pi ^{4} \\ & \cong -36.0087 , \end{aligned}$$

$G''_{4}(s)<0$ and $G'_{4}(s)$ is strictly decreasing for $1/4 < s < \pi^{2}/4$. Since $G'_{4}(1/4) = 4(-900+1740 \pi ^{2}-501 \pi ^{4}+122 \pi ^{6}-9 \pi ^{8}) \cong -2544.56$, $G'_{4}(s)<0$ and $G_{4}(s)$ is strictly decreasing for $1/4 < s < \pi^{2}/4$. Therefore, we have $G_{2}(t) > G_{4}(\pi^{2}/4) =48 \pi^{6}$ for $1/2< t< \pi/2$. By the squeeze theorem, $F_{1}(t) >16$ for $1/2 < t< \pi/2$. Also, we have

$$\begin{aligned} F_{1}(t) < \frac{G_{2}(\frac{1}{2})}{3\pi^{6}} \end{aligned}$$

for $1/2 < t <\pi/2$ and

$$\begin{aligned} F_{1}(0+)-\frac{G_{2}(\frac{1}{2})}{3\pi^{6}} &=\bigl(\pi^{2} -4 \bigr)^{2} -16 -\frac{G_{2}(\frac{1}{2})}{3\pi^{6}} \\ & = \bigl(\pi^{2} -4 \bigr)^{2} -16 -\frac{-300 +840 \pi^{2}-327 \pi^{4} -163 \pi^{6}-5 \pi^{8} +3 \pi^{10}}{3\pi^{6}} \\ & =\frac{300-840 \pi^{2} +327 \pi^{4} +163 \pi^{6} -19 \pi^{8}}{3\pi^{6}} . \end{aligned}$$

By $300-840 \pi^{2} +327 \pi^{4} +163 \pi^{6} -19 \pi^{8} \cong 286.654$, we have

$$\begin{aligned} F_{1}(0+)>\frac{G_{2}(\frac{1}{2})}{3\pi^{6}} . \end{aligned}$$

Thus, we can get $16 < F_{1}(t) < F_{1}(0+)$ for $0< t<\pi/2$. The proof of Theorem 2.1 is complete. □

### Proof of Theorem 2.2

#### Proof of Theorem 2.2

We have

$$\begin{aligned} F_{1}(x)&= \frac{\pi^{2} x}{4 +\sqrt{(\pi^{2} -4)^{2} + (2\pi x)^{2}}} -\frac{8 x}{3 +\sqrt{25 + \frac{80}{3} x^{2}}} \\ & = \frac{x ( -96 +9 \pi^{2} +\sqrt{15} \pi^{2} \sqrt{15 +16 x^{2}} -24 \sqrt{16-8 \pi^{2} +\pi^{4} +4 \pi^{2} x^{2}} )}{ (9 +\sqrt{15} \sqrt{15+16 x^{2}} ) (4 +\sqrt{16-8 \pi^{2}+\pi^{4}+4 \pi^{2} x^{2}} )} \\ & = \frac{x F_{2}(x)}{ (9 +\sqrt{15} \sqrt{15+16 x^{2}} ) (4 +\sqrt{16-8 \pi^{2}+\pi^{4}+4 \pi^{2} x^{2}} )} . \end{aligned} $$

The derivative of $F_{2}(x)$ is

$$\begin{aligned} F_{2}'(x) &= \frac{16 \pi^{2} x (-6 \sqrt{15+16 x^{2}}+\sqrt{15} \sqrt{16-8 \pi^{2} +\pi^{4} +4 \pi^{2} x^{2}} )}{\sqrt{15+16 x^{2}} \sqrt{16-8 \pi^{2} +\pi^{4} +4 \pi^{2} x^{2}}} \\ &= \frac{16 \pi^{2} x F_{3}(x)}{\sqrt{15+16 x^{2}} \sqrt{16-8 \pi ^{2}+\pi ^{4}+4 \pi ^{2} x^{2}}} . \end{aligned}$$

Here, we have $15 (16-8 \pi ^{2}+\pi ^{4}+4 \pi ^{2} x^{2} ) - 36(15+16 x^{2} ) = 3 (-100-40 \pi ^{2}+5 \pi ^{4}-192 x^{2}+20 \pi ^{2} x^{2} )$. Since $-192 +20 \pi^{2} >0$ and $-100-40 \pi ^{2}+5 \pi ^{4}-192 x^{2}+20 \pi ^{2} x^{2}=0$ for $x=\sqrt{\frac{100+40 \pi ^{2}-5 \pi ^{4}}{20 \pi ^{2}-192}} \cong 1.198$, we have $F_{3}(x)<0$ for $0< x<\sqrt{\frac{100+40 \pi ^{2}-5 \pi ^{4}}{20 \pi ^{2}-192}}$ and $F_{3}(x)>0$ for $x>\sqrt{\frac{100+40 \pi ^{2}-5 \pi ^{4}}{20 \pi ^{2}-192}}$. Therefore, $F_{2}(x)$ is strictly decreasing for $0 < x < \sqrt{\frac{100+40 \pi ^{2}-5 \pi ^{4}}{20 \pi ^{2}-192}}$ and strictly increasing for $x > \sqrt{\frac{100+40 \pi ^{2}-5 \pi ^{4}}{20 \pi ^{2}-192}}$. From $F_{2}(0+)=0$ and

$$\begin{aligned} F_{2}(\alpha) &= -96+9 \pi^{2} +\sqrt{15} \pi^{2} \sqrt{15+16 \alpha^{2} } -24 \sqrt{16 -8 \pi^{2} + \pi^{4} +4 \pi^{2} \alpha^{2}} \\ &= -96+9 \pi^{2} +\sqrt{15} \pi^{2} \biggl( \frac{\sqrt{15} (112-11 \pi ^{2} )}{-48+5 \pi ^{2}} \biggr) -24 \biggl( \frac{192+32 \pi ^{2}-5 \pi ^{4}}{-48+5 \pi ^{2}} \biggr) \\ &= 0 , \end{aligned}$$

we can get $F_{2}(x)>0$ for $x> \alpha$ and *α* is the best possible. The proof of Theorem 2.2 is complete. □

### Proof of Theorem 2.3

#### Proof of Theorem 2.3

We have

$$\begin{aligned} F_{1}(x) &= \frac{8 x}{3 +\sqrt{25 + \frac{256}{\pi^{2}} x^{2}}} -\frac{\pi^{2} x}{4 +\sqrt{32 + (2\pi x)^{2}}} \\ &= \frac{\pi x (32-3 \pi^{2} -\pi \sqrt{25 \pi^{2} +256 x^{2}}+16 \sqrt{8 +\pi^{2} x^{2}} )}{ 2 (3 \pi +\sqrt{25 \pi^{2} +256 x^{2}} ) (2+\sqrt{8+\pi^{2} x^{2}} )} \\ &= \frac{\pi x F_{2}(x)}{ 2 (3 \pi +\sqrt{25 \pi^{2} +256 x^{2}} ) (2+\sqrt{8+\pi^{2} x^{2}} )} . \end{aligned}$$

The derivative of $F_{2}(x)$ is

$$\begin{aligned} F_{2}'(x) &= \frac{16 \pi x (\pi \sqrt{25 \pi^{2} +256 x^{2}} -16 \sqrt{8+\pi^{2} x^{2}} )}{\sqrt{25 \pi^{2} +256 x^{2}} \sqrt{8+\pi^{2} x^{2}}} \\ &= \frac{16 \pi x F_{3}(x)}{\sqrt{25 \pi^{2} +256 x^{2}} \sqrt{8+\pi^{2} x^{2}}}. \end{aligned} $$

Since $\pi^{2} (25 \pi^{2} +256 x^{2}) - {16}^{2} (8+\pi^{2} x^{2}) = -2048 +25 \pi^{4} \cong 387.227$, we can get $\pi^{2} (25 \pi ^{2}+256 x^{2}) > {16}^{2} (8+\pi ^{2} x^{2}) $ for $x>0$. Therefore, $F_{3}(x)>0$ and $F_{2}'(x)>0$ for $x>0$. Since $F_{2}(x)$ is strictly increasing for $x>0$ and

$$\begin{aligned} F_{2}(\beta) &= 32-3 \pi^{2} -\pi \sqrt{25 \pi^{2} +256 \beta^{2}} +16 \sqrt{8 +\pi^{2} \beta^{2}} \\ &= 32-3 \pi^{2} -\pi \biggl( \frac{512 +96 \pi^{2} -17 \pi^{4}}{\pi ( -32 +3 \pi^{2} )} \biggr) +16 \biggl( \frac{192-12 \pi^{2} -\pi^{4}}{2 (-32+3 \pi ^{2} )} \biggr) \\ &= 0 , \end{aligned}$$

we can get $F_{2}(x)>0$ for $x> \beta$ and *β* is the best possible. The proof of Theorem 2.3 is complete. □

### Proof of Theorem 2.4

#### Lemma 2.6

*For* $x>0$, *we have*

$$\begin{aligned} \frac{75600 \pi ^{2} x}{\sqrt{8+\pi ^{2} x^{2}}}+\frac{64 \pi ^{2} x^{7}}{\sqrt{8+\pi ^{2} x^{2}}} >\frac{25200 \sqrt{15} \pi ^{2} x}{\sqrt{15+16 x^{2}}} . \end{aligned}$$

#### Proof

We have

$$\begin{aligned} \biggl( \frac{75600 \pi ^{2} x}{\sqrt{8+\pi ^{2} x^{2}}}+\frac{64 \pi ^{2} x^{7}}{\sqrt{8+\pi ^{2} x^{2}}} \biggr)^{2} - \biggl( \frac{25200 \sqrt{15} \pi ^{2} x}{\sqrt{15+16 x^{2}}} \biggr)^{2} &= \frac{256 \pi ^{4} x^{2} F_{1}(x)}{ (15+16 x^{2} ) (8+\pi ^{2} x^{2} )} , \end{aligned}$$

where $F_{1}(x)= 37209375+357210000 x^{2}-37209375 \pi ^{2} x^{2}+567000 x^{6}+604800 x^{8}+240 x^{12}+256 x^{14}$. Here, we have

$$\begin{aligned} F_{1}(x) & > 37209375+357210000 x^{2}-37209375 \pi ^{2} x^{2}+567000 x^{6} \\ & = 70875 \bigl(525+5040 x^{2}-525 \pi ^{2} x^{2}+8 x^{6}\bigr) . \end{aligned}$$

We set $t=x^{2}$ and $F_{2}(t) = 525+5040 t -525 \pi^{2} t +8 t^{3}$, then the derivative of $F_{2}(t)$ is $F'_{2}(t) = 5040-525 \pi^{2} +24 t^{2}$. Since $F'_{2}(t)=0$ for $t= \frac{1}{2} \sqrt{\frac{1}{2} (-1680+175 \pi ^{2} )} \cong 2.4285$, we have $F'_{2}(t)<0$ for $0< t< \frac{1}{2} \sqrt{\frac{1}{2} (-1680+175 \pi ^{2} )}$ and $F'_{2}(t)>0$ for $t>\frac{1}{2} \sqrt{\frac{1}{2} (-1680+175 \pi ^{2} )}$. Hence,

$$\begin{aligned} F_{2}(t) & \geq F_{2} \biggl(\frac{1}{2} \sqrt{ \frac{1}{2} \bigl(-1680+175 \pi ^{2} \bigr)} \biggr) \\ & = \frac{35}{2} \bigl(30+48 \sqrt{70 \bigl(-48+5 \pi ^{2} \bigr)}-5 \pi ^{2} \sqrt{70 \bigl(-48+5 \pi ^{2} \bigr)} \bigr) \\ & \cong 295.843 \end{aligned}$$

for $t>0$. Therefore, $F_{1}(x) >0$ and the proof of Lemma 2.6 is complete. □

#### Proof of Theorem 2.4

We have

$$\begin{aligned} F_{1}(x) &= \frac{8x +\frac{32 x^{7}}{4725}}{3+\sqrt{25 +\frac{80}{3}x^{2}}}-\frac{\pi ^{2} x}{4 +\sqrt{32 +(2 \pi x)^{2}}} \\ &= \frac{x F_{2}(x)}{3150 (9 +\sqrt{15} \sqrt{15+16 x^{2}}) (2 +\sqrt{8 + \pi^{2} x^{2}})} , \end{aligned}$$

where $F_{2}(x) =151200-14175 \pi^{2} +128 x^{6} -1575 \sqrt{15} \pi^{2} \sqrt{15+16 x^{2}} +75600 \sqrt{8 +\pi^{2} x^{2}} +64 x^{6} \sqrt{8 +\pi^{2} x^{2}}$. The derivative of $F_{2}(x)$ is

$$\begin{aligned} F_{2}'(x)& = 768 x^{5}-\frac{25200 \sqrt{15} \pi^{2} x}{\sqrt{15+16 x^{2}}}+ \frac{75600 \pi^{2} x}{\sqrt{8 +\pi^{2} x^{2}}}+\frac{64 \pi^{2} x^{7}}{\sqrt{8 +\pi^{2} x^{2}}}+384 x^{5} \sqrt{\pi^{2} x^{2}+8} \\ &> -\frac{25200 \sqrt{15} \pi^{2} x}{\sqrt{15 +16 x^{2}}}+\frac{75600 \pi^{2} x}{\sqrt{8 +\pi^{2} x^{2}}}+\frac{64 \pi^{2} x^{7}}{\sqrt{8 +\pi^{2} x^{2}}} . \end{aligned}$$

By Lemma 2.6, we have $F'_{2}(x)>0$ and $F_{2}(x)$ is strictly increasing for $x>0$. From $F_{2}(0+)=37800 (4 +4 \sqrt{2} -\pi^{2} ) \cong -8041.96$, $F_{2}(\gamma)=0$ and $F(\infty)=\infty$, we can get $F_{2}(x)>0$ for $x>\gamma$. The proof of Theorem 2.4 is complete. □

## Conclusions

In this paper, we established some inequalities involving arctangent. The double inequality in Theorem 2.1 provides sharper quadratic estimations than (1.2) and (1.3) for a location away from zero. By Theorems 2.2, 2.3 and 2.4, we obtained Proposition 2.5 immediately.
